# Delineating the relationship between maritime insecurity and COVID-19 pandemic on West African maritime trade

**DOI:** 10.1186/s41072-022-00121-w

**Published:** 2022-08-08

**Authors:** Anthony Djaba Sackey, Bernard Lomotey, Abigail Dede Sackey, Raphael Ofosu-Dua Lee, Abraham Akwetey Teye, Richmond Kennedy Quarcoo, John Bansah

**Affiliations:** 1 Bureau Veritas Group Marine and Offshore, Accra, Ghana; 2 DNV Maritime, Ben Marine Services, Accra, Ghana; 3 German Institute of Technology, Accra, Ghana; 4 DNV GL Oil and Gas/Grassfield Maritime Consultants Lagos, RROC Industrial Limited, Accra, Ghana; 5 Ghana Navy, Naval Headquarters Burma Camp, Accra, Ghana; 6 Plastic Punch Organisation, Accra, Ghana; 7 Mega Food Industries, Khartoum, Sudan

**Keywords:** Gulf of Guinea, Maritime insecurity, Piracy and blue economy, Shipping route, Africa continental free trade area, West Africa maritime corridor

## Abstract

In this paper, three steps are made. First, an effort is made to show a consequential effect of maritime insecurity on seafarers and marine professionals; they are at risk of a complicated string of processes that impact their lives. Second, there is the risk to the environment and property. Third, the economic cost of traditional maritime crimes is examined against the potential maritime trade expansion from implementing the African Continental Free Trade Area (AfCFTA). Third, the policy and regulatory measures implemented in the region against piracy are assessed to propose additional measures for improvement. Essentially, the study deploys a case study approach with a three-year field observation over the Gulf of Guinea region and is supported by outcomes of various remote interviews, in addition to online surveys conducted over three months—findings are juxtaposed with the estimated cost of piracy and potential implications for policies driving economic advancement. The results showed inadequate maritime surveillance despite enormous legal frameworks amidst the current structures of regional and international corporations. The piracy cost is high and inevitable as a factor of insurance coverage passed onto end consumers. Response to piracy has been reactionary rather than proactive, as attacks have continued in territorial and offshore areas into 2021. The impact will be visible on AfCFTA post-COVID-19. The study highlights the need for a community-based approach to surveillance modelled after the community policing model currently implemented in Ghana.

## Introduction

As a continuation of a preceding paper,^1^ the current paper also examines maritime security threats of piracy and armed robbery against the economic prosperity and livelihood of citizens in West Africa. As with the previous paper, the concerns here were properly denoted and dissected with the expectation of addressing concerns with the most appropriate recommendations based on an integrated approach. Here, efforts are made to understand the economic cost of piracy and armed robbery driving the current dynamics in security measures being implemented.

Security, as a generic term, fundamentally “*has to do with the presence of peace, safety, gladness, and the protection of human and physical resources or absence of crisis or threats to human dignity, all of which facilitate the development and progress of any human society*” (Afolabi 2015). William (2008), on the other hand, submits that security was primarily associated with alleviating threats to cherished values. Thus, threats that threaten the survival of a particular reference object are critical. Afolabi further noted that the term’s meaning remains ambiguous as its scope expands daily, given the changing nature of physical and nonphysical threats within society. He further alluded to the seven dimensions of human security: economic, environmental, food, health, community, political, and personal security, which remains a genuine concern within the maritime sector. Therefore, Afolabi suggested that the security concept’s elastic nature has attracted different meanings and varying opinions. Undoubtedly, the concept of security for decades has transformed into a preoccupation. Therefore, in this vein of broadness, the context of maritime security and insecurity is discussed amidst underlining the economic limitations and implications of West African nations.

Noting the current expansion of the African Continental Free Trade Area Agreement (AfCFTA) in terms of signatories with the willingness to trade regionally as bound by courage amid and post COVID-19 restrictions in 2020, according to the International Chamber of Commerce International Maritime Bureau (ICCIMB) (2020), the problem of maritime insecurity in the West African subregion had not shown any sign of slowing down (ICCIBM 2020).

These thus subject the economic prospects aimed at an expanded view of industrialisation and overall trade value in terms of market share amongst Africans on continents to a window of uncertainty (risk), given the poor and inadequate nature of road networks and railway lines connecting countries in the south to the north, from west to the east and vice versa. There is no doubt that the maritime trade route across the continent remains the best bet to reach coastal and landlocked nations with goods. This can be done with little to no new effort, given that major seaports’ facilities across the continent are developed and equipped with state-of-the-art machinery and can handle all forms of cargo shipment. However, the successful implementation of the AfCFTA will require additional investment in marine transport assets such as cargo barges, cargo merchant vessels, and passenger carriers by individuals and institutions. Whereas the maritime corridor as a means of heavy transport remains a viable alternative to be leveraged in advancing the AfCFTA goals, the age-old concern of political instability (Otieno 2008) and maritime insecurity within sections of the continental trade routes continues to alarm investors.

The two-part study’s objective set and discussed in paper one (Sackey et al. 2022) continues to drive the focus of this paper by showing the consequential effect of maritime insecurity on the lives of seafarers and marine professionals (Raunek 2021; Caesar et al. 2015). It also impacts the problem on the environment and property or maritime assets. In this paper, the economic cost of maritime insecurity in the region and the consequential impact on the potential maritime trade expansion arising from the successful implementation of the AfCFTA are further highlighted and detailed. The concern for maritime insecurity amid AfCFTA implementation is evaluated against current policy and regulatory measures of some major nation-states and regional blocks, as noted in paper one as being implemented locally and subject to the added measures. The paper also recognises the COVID-19 economic impact on the maritime industry and personnel worldwide and in Africa and how the situation has played with the rising concern of maritime insecurity in various regions. Therefore, the method and analysis believed to be the most suitable for developing the knowledge, based on the current economic cost and trends, problems, regulations, and policies, thus well espoused in paper one, is deployed herein to promote a sustainable blue economy. Rhetorically, what is the economic cost of piracy to trade in Africa and Africa as a whole? This question drives the study’s expectations in line with the implications for AfCFTA.

Concerning the impact, the study is expected to highlight the economic implications of growingly unchecked piracy concerns, its uncertainties regarding the noble implementation of the AfCFTA industrialisation, and the upscale economic policy, lives, and livelihood of maritime professionals in the industry. Essentially, the outcomes will project an understanding of the concerns that burden the potential benefit of the success of the AfCFTA program from a maritime perspective by attempting to answer the pertinent questions. It also delineates any gaps in the current national and local regulations and the AfCFTA policy, where the potential for further research to improve the effectiveness can be recommended.

### Detailed summary from paper one

In order to proceed with the literature review, this sub-section summarises Paper One since it is the foundation for the various discussions addressed herein. The paper examines the literature on the current maritime security threat in West Africa amidst the COVID-19 pandemic to identify the most appropriate recommendations with an integrated approach to tackling piracy. In addition, it attempts to identify and investigate the current policy implementations and practices regulating maritime security in the subregion. Again, the impact of the COVID-19 crisis on security will also be slightly examined. Thus, establishing the relationship for each impact on trade links to various measures implemented nationally and regionally.

Concerning trade and associated concerns, Paper One’s findings recognise that the Gulf of Guinea (GoG) region remains a critical resource for the socioeconomic development of the countries of West Africa. The continued expansion of maritime infrastructure over the decade on the heels of trade expansion is linked to AfCFTA’s potential. The paper asserted that the success or failure of the AfCFTA vision would gradually be tied to the trend of maritime insecurity if the situation is allowed to persist. As of 2019, Africa accounted for 12 per cent and 7 per cent of the world’s maritime transport trade volumes loaded and unloaded within developing countries. However, this is expected to increase drastically in intra-African nations’ trade across the continent. The paper affirmed UNCTAD’s (2020) call for the need to address the challenges in the sector that relate to maritime insecurities amid the changing dynamics in the current local, national and regional connectivity needs of the entire continent.

The paper also defined ‘maritime insecurity’ based on Limo’s (2012) definition of maritime security since there is currently no standardised definition in the industry. This was primarily due to cataloguing the nature of the maritime threat and its evolution over the years. According to various data from the ICC IBM, the GoG region has become a hotspot for traditional piracy and armed robbery maritime crimes. The same data also highlighted the urgency of the current threat to the region’s socioeconomic and political stability. The paper notes that the importance and urgency of maritime security cannot be overemphasised. The rippling effect of these challenges translates into rising commodity pricing, among other concerns. These are cascading effects on the cost of insurance premium cover, particularly kidnap and ransom (K&R).

Nonetheless, the paper further suggests that piracy costs in the region are subjective. It cited Nigeria's case, pegged at over $1.1 billion in 2020. According to Paper One, average ransoms per crew member were capped at US$50,000. However, there were no sufficient data to assist in the determination of estimated economic costs within the GoG region; thus, the paper highlighted the need for such an undertaking against AfCFTA’s implementation.

Concerning implementation policies and regulations, the paper recognises that the efforts to curb the menace have been romped up amid policy strategies, including local, regional and international collaborations, in terms of capacity building, funding and technological support. An example is the enforcement of legislative instruments under the *Yaoundé Code of Conduct*.

Concerning enforcement, the paper further recognises that enforcement alone will remain ineffective and reiterates calls for national governments to promote the socioeconomic wellbeing of their citizens within vulnerable communities to serve as a disincentive for the unemployed seeking to join criminal syndicates. While efforts towards successful enforcement and prevention of maritime crimes such as piracy and robbery heavily depend on credible information sharing, informant credibility, and cooperation, the paper recognises that little to no effort has been made to ensure that such cooperation or official collaboration exists between the enforcement institutions, such as the national navies from the region and the fishermen as well as fishmongers of the adjoining coastal communities. Contrary, the paper observes that the fishermen from these communities are most likely forced into collaborating with these criminal syndicates who induce them with threats or economic rewards.

The rise in maritime crimes in the GoG area is a simple indication that the approach taken over the last decade to redress the problem is either insufficient and/or more needs to be done. The paper highlights the first-ever maritime crime prosecution in the region against enforcement. This is a piece of welcoming news that serves as a deterrent. While maritime crime in the region is not limited to the traditional crimes of piracy and theft, policy interventions have also increasingly focused on the crimes and malpractices in the fishing industry that are believed to be the reason for the depleting fishing stock in the region (Sackey et al. 2022).

## Literature review

The subsections that follow discuss the worldwide concerns of maritime security amid and post-COVID-19 in general. It, however, lays a narrow focus on piracy within the West African subregion. Thus, the economic implications of nation-states’ efforts should be established, as they project to capitalise on intra-trading via AfCFTA on the continent and beyond through its maritime corridors.

### Overview maritime security and the matters arising

In discussing the concept of maritime security or insecurities, we first revisit and examine the basic term security espoused in the introductory section and what it does imply in national sovereignty, maritime operations, the environment, society, and economics.

According to Pichon and Pietsch (2019), the traditional meaning of maritime security is exhumed from national defence policy, where the concept of maritime security connotes the safeguarding of national territory from attacks using the sea. Similarly, the term addresses issues relating to illegal dumping of toxic waste in territorial waters regarding the environment, issues of human trafficking in the social context, and issues of illegal fishing economically. Notwithstanding this, no international consensus regarding maritime security definition has been reached (Pichon and Pietsch 2019). Therefore, from the broader scope of security concerns (or insecurities), maritime security, according to Klein (2015), can be envisaged as the “*protection of a state’s land and maritime territory, infrastructure, economy, environment and society from certain harmful acts occurring at sea*”.

Liwång (2017) also thus notes that regarding maritime insecurities, piracy remains one of the most frequent threats observed, although measures are in place to have it reduced. In comparison to other maritime safety matters, it is the least investigated concern. However, concerning piracy trends in Africa over the years, piracy off the coast of Somalia remains the most investigated case (Liwång 2017). According to Liwång, those results cannot necessarily be generalisable, as piracy off the coast of West Africa has “*been shown to be more diverse, successful and dangerous*”.

Therefore, Pristrom et al. (2016) parroted that the pirates’ operation mode (modus operandi) off Somalia was to attack ships while under full speed with the intent of hijacking the ship or kidnapping crew for ransom, compared to the historic petty theft of crew personal effects and ships stores observed in West Africa. Thus, current piracy trends in West Africa have transformed from near coast concerns into a more sophisticated and diverse criminal activity with violent tendencies (Pristrom et al. 2016) and incidences reported beyond 100 nautical miles out on the high seas (ICCIBM 2020) against ships at anchor or drifting while waiting for cargo or orders, and of ship underway. At least three varying intents are ascribed to criminal activity: armed robbery, cargo theft, and kidnapping (Pristrom et al. 2016).

In late 2019, Pichon and Pietsch (2020) asserted in their study that the most trending form of maritime piracy and armed robbery observed within the GoG region was the hijacking of ships with the intent of kidnapping for ransom payments. Pichon and Pietsch further reiterated that the economic cost of GoG piracy estimated for 2017 was over €750 million. Thus, after long neglect, GoG states are becoming increasingly abreast of the status of their maritime security, which directly and indirectly has a global dimension in terms of impact. Therefore, maritime crime within the GoG region remains a global concern. Furthermore, the root causes of such crimes at sea reoccurrence are found on land rather than on the high seas (Pichon and Pietsch 2020). The economic implications are further discussed in the proceeding section.

### Economic cost of piracy and armed in the Gulf of Guinea area

The 2020 reports confirm that the piracy menace within the GoG cost Nigeria over $1.1 billion (Support 2021). It also suggested that average ransoms of $50,000 per crew member are now a reality following successful kidnappings of crews and thus form the most lucrative piracy strategy since the 2014 oil price crash (GM (Great Minds) Event 2021). The activities of pirates in the GoG area have affected various vessel types, including fishing vessels, tankers, bulk carriers, and container vessels. The estimated economic cost within the GoG region remains scant, as insufficient data exist for a wholesome estimate (The Critical Maritime Routes Monitoring, Support and Evaluation Mechanism (CRIMSON III) 2021). However, such a cost is essential in evaluating piracy’s economic impact on the region and the world.

In the related region of the Gulf of Arden, Somalia, according to Besley et al. (2012, 2019), for every $120 million seized by Somalia pirates, a cost of $0.9 and $3.3 billion is passed to end consumers of the shipping industry. Thus, observations from 2008 data led to an approximately 8% increase in shipping costs. According to Mueller et al., this is enough money to help advance the employment of well over a million Somalis for a whole year. The European Commission (EC 2013) estimated that over 13,558 ships were routed within the GoG region within just six months of the previous year, 2012. The shipping traffic has continued to rise along with the major macroeconomic indicators of the nations’ states found in the area.

Nonetheless, the year 2021, within the GoG area, has seen a continuation of pirate attacks. However, per Dryad’s analysis remains slightly low compared to the same period last year, with the latest incident involving the commercial fishing vessel, the Iris S, operating from the Ghanaian port of Tema. It was reported to have been boarded approximately 100 nautical miles away to the south of Cotonou, Benin. With a crew size of 36, the 500 gross tonnage vessel is assessed by experts as an easy target for the pirates. Thus, due to her age (The Maritime Executive 2021a, 2021b). Economically, these uncertainties influence insurance coverage rates, such as war risk and kidnapping and ransom, K&R (Bowden et al. 2010), which are subsequently passed onto end consumers.

### AfCFTA considerations and the need to boost economic recovery post-COVID-19

Since creating the African Continental Free Trade Area, AfCFTA, Africans earnestly look forward to the many economic opportunities that the free trade zone promises. This is partly because of the massive unemployment, huge youth population, limited industries, and lack of finance. The AfCFTA initiative is expected to capitalise on a liberalised market of over 1.2 billion people, with a combined gross domestic product (GDP) of US$3 trillion. Successful implementation translates into Africa’s manufacturing sector doubling in size, with annual output increasing to $1 trillion by 2025 (Signé, 2019).

According to the president of Ghana, who at the time also chaired the African Union (AU), the trade zone created is “*the world’s largest free trade area since the formation of the World Trade Organisation*” (Communications American Chamber of Commerce-Ghana, AMCHAM, 2021). This assertion has also been reiterated by many others including Caroline Kende-Robb, a senior fellow of the African Center for Economic Transformation (ACET) in an article with the World Economic Forum (Kende-Robb 2021). The president emphasised this assertion when he stressed his goal of making Ghana the trade hub within Africa. He did so, decrying any efforts of the international economic order, which does not fully inure to the benefits of Africa (Citinews 2021; ghfactsonline.com 2020). African leaders today recognise that the potential industrial advance may not be realised without a play on regional integration and development of trade as they pool resources towards AfCFTA’s realisation. Juxtaposing the aims of the AfCFTA framework to the long-standing industrialisation policy agenda in Africa, which mostly has failed to materialize, AfCFTA has been overwhelmingly received by all nation-states of the union with a strong desire to work together. Studies suggest a historical tendency towards deindustrialization in Sub-Saharan Africa (Lungu 2019; Jalilian and Weiss 2000). Thus, a constant decline in manufacturing and industrial value addition shares has continued in recent years (Lungu 2019; Aryeetey and Moyo 2011).

This long-running African problem is expected to seize as AfCFTA takes effect in unlocking the potential for enhanced regional and continental economic integration. It is also expected to support industrialisation by facilitating the movement of people (human capital) and service availability to this effort. Essentially, AfCFTA ensures that AU’s existing framework on industrial policy growth (termed agenda 2063 with expected growth of 10 per cent) is realised. Industrialisation strategies or policy frameworks have been adopted at the Member State level. This includes countries like Djibouti, Egypt, Eritrea, Ethiopia, Gabon, Kenya, Lesotho, Liberia, Mauritius, Nigeria, Rwanda, Tanzania, Uganda, and Zimbabwe (Asamenew 2019).

The expected economic outburst following a successful implementation of the AfCFTA agreement implies that a significant amount of consumer goods and services will be exchanged across the length and breadth of Africa based on the various modes of transport currently available. Tramp shipping across Africa thus has growth potential and is best placed to oversee large shipments of goods across Africa on the seafront compared to truck shipment. Therefore, the safety and security of Africa’s major sea trading routes must be fortified against armed robbery and piracy (GM Events 2021).

### Repositioning Africa for high earn return in maritime trade and manpower needs

The growing call for inclusiveness in all sectors of the world economies of minorities in 2020 should have placed Africa’s non-traditional maritime labour force at the centre of attention amid the COVID-19 health and economic crises. Instead, attention has focused on the rising nefarious activities of pirates and armed robbers in the GOG, where every local and international resource is utilised to combat the piracy and armed robbery menace (GM Event 2021).

As mentioned in the earlier “AfCFTA considerations and the need to boost economic recovery post‑COVID‑19” section, the growing continental trading expected among African nations presents the potential for growth in the liner and tramp shipping industry (STOCKCARGO 2021). In Africa, tramp ships (mostly constituted by tugboat towing barges stacked with containers load for delivery at various locations via inland waters) remain few in the GoG trading area, servicing the liner container vessels (Kouassi 2018), bulk, and liquid bulk carriers (of energy industry), particularly LPG, LNG, and crude oil transport. However, the acquisition of ships and trucks for intermodal transport, the construction and expansion of ship repair yards, warehousing, and the continuous engagement of financial, legal, insurance (particularly war risk and K&R) and maritime institutions promise a high earn maritime trading value. Therefore, this is an opportunity to utilise Africa’s training institutions and growing maritime labour force. Such an opportunity is at crossroads with the growing maritime insecurity of the region (GM Event 2021).

### Ending maritime insecurity for economic advancement: a newer approach to policing the maritime domain of West Africa

In the last couple of years, the fight against maritime insecurity in the region has come under severe scrutiny due to the rising incidence of piracy in the region. This has seen the implementation of various efforts from local authorities and international partners. According to Azumah et al. (2019), the roles of informants are critical for effective crime control. In other words, deriving information at any time is critical when dealing with a rise in crime. While it remains unclear what model of maritime policing is in place within the region, this has not been clearly defined and explained. However, current trends suggest that the current regimes of simple naval patrols and waiting to respond to a broadcast of maritime crime at sea on radio poorly deter such crimes. The increasing presence of naval vessels in the region partly supported by international partners is still not sufficient for the total sea coverage of the area. Therefore, a newer approach should breed some progress. While the fighting of crime generally, as explained by Azumah et al. (2019), cannot be achieved without communication and collaboratory efforts, the maritime domain of west and central Africa continues to receive such support, albeit disjointed. The situation indeed calls for a newer approach like the community policing model (Ghana Police Service, GPS 2017).

The GPS, in its quest to find ways to “*enhance collaboration and communication with local communities*”, established in June 2002 a new unit referred to as the Community Policing Unit (CPU) (GPS 2017). The GPS observed that the credibility of information received, and the informant was intertwined with the service’s ability to maintain positive community relations. They asserted that this was essential for the police’s continuous reliance on informants’ information on crime. Furthermore, they viewed the approach as the future of policing since it projected a more inclusive approach to crime-fighting.

In Ghana, the CPU served as a focal platform for strategy development regarding the design and implementation of community policing throughout Ghana (GPS 2017). Hence, involving community residents in policing imparts a sense of ownership, willingness, and personal responsibility for the community’s safety and security. Therefore, the police can implement crime prevention measures through this two-sided exchange with the community’s assistance (GPS 2017). Azumah et al. (2019) buttressed these concerns. They alluded in their study that informants were relatively fewer where local communities are of concern. These informants also believed reporting crimes to the police was critical to fighting crime. They recognise the dangers their lives may be exposed to as they act in such roles to make their native societies safe (Azumah et al. 2019).

Azumah et al. (2019) concluded that the protection of informants heavily relies on a good relationship between community members and police. They also recommended that the Government and GPS provide its officers with a good blend of language diversity since effective communication is fundamental to positive police-community relationships. There is also a need for in-service training by the government and GPS to improve community policing and trust-building (Azumah et al. 2017). Similarly, the navies of nation-states in the region are expected to maintain a crime-free domain for fishing communities, shippers, shipowners, and many other stakeholders. The navy policing, in this case, could make use of the community policing model discussed herein.

## Methodology

The study deploys a mixed qualitative and quantitative case study approach while considering the effect of maritime security concerns on the general seafaring population worldwide, with a particular focus on the West African trade route. This was essential when developing the population sample size and the research design, deciding on the most appropriate instrument to be used in the data collection process and adopting the most effective technique for validating data reliability. Therefore, a case study of a mixed-method survey and field observation deployed follows a three-phase approach. The approach is slightly modified based on Kwabia’s (2006) and Sackey et al.’s (2021a, b) methods. The successful deployment of this method thus helps provide the needed understanding, sensitivity, and emotional framework capable of capturing the current threats faced by the seafaring community and stakeholders alike.

### Study area

**Fig. 1:**
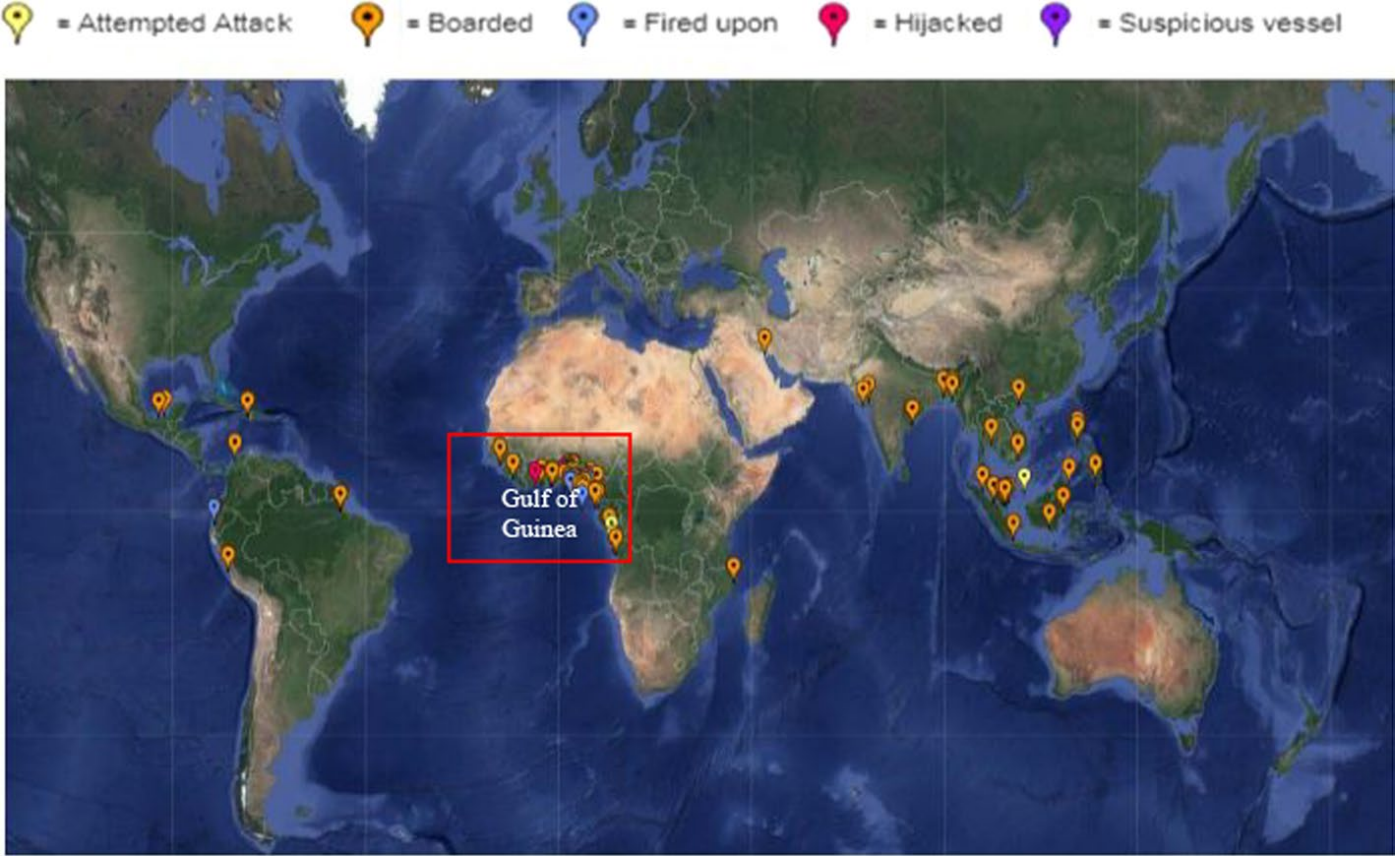
Map showing regional high sea pirate and armed robbery attacks across Africa, with particular emphasis on the West Coast (Gulf of Guinea). *Source*: Courtesy ICC IBM (2014, 2020)

The GoG area chosen and examined in this study considers the ICC IBM piracy and armed robbery risk map of 2014 (see Fig. 1 courtesy ICCIBM (2014) pirate and armed robbery map) in identifying the hotspots and mapping out the maritime corridor of interest. However, noting that the entire GoG region—currently exploding with a rising incidence of pirate attacks (ICCIBM 2020) near the coast and expanded deep-sea hydrocarbon field developments (Sackey 2021)—shows no sign of slowing, the current trend of pirate attacks often has targeted commercial trading vessels across the region. The study, therefore, focuses on shipping routes, offshore installation sites and port anchorages where reported incidences have been documented as targeted sights in certain situations (UNODC 2020).

As seen in Fig. 1, the sea coverage stretches over approximately 6,000 km of coastline from Senegal northwest to Angola southwest of the region (covering 11,000 square kilometres (4,247 sq. miles) of sea area). It remains a vital shipping zone transporting oil, gas, and goods to and from central and southern Africa. The tropical nature of the area generally informs the nature of weather, which is characterised by high levels of visibility (mostly 10 nautical miles) and occasional low visibility, wave heights averaging from 1 and 3 m and ocean surface currents primarily set in the southwest quadrant direction (Sackey 2021). Thus, weather patterns across seasons are generally favourable for marine navigation for all sizes of ships and watercraft.

### Research design and study scope

The three-phase descriptive design of the study examines seafarers’ experiences via survey questionnaires and observation of operations and concludes with the final survey of experts. The choice of this design style was to understand better the relationship between observed concerns, perceptions, and direct experiences of maritime professionals regarding maritime security incidents, policies, and practices. The study’s first phase describes a period spent onboarding various marine vessels during operations in the GoG region, thus observing some of the safety protocols instituted on the vessels in compliance with the International Ship and Port Security, ISPS Code. The study's second phase involved surveying seafarers whose ships regularly transited the region to gain insight into their experiences. Finally, the third phase of the study was achieved by sampling the knowledge of some designated statutory maritime personnel (experts) and the experience of a maritime crime victim.

**Fig. 2:**
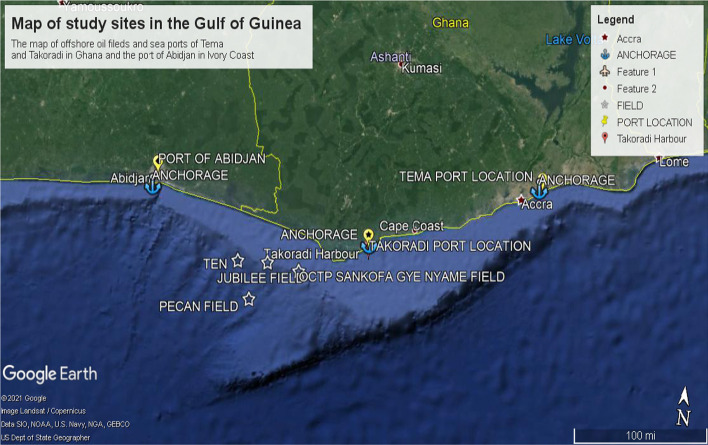
Map showing various offshore and marine port facilities surveyed during the study between Ghana and the Ivory Coast in the Gulf of Guinea. *Source*: Courtesy Google Satellite (2021)

Phase one of the study, which deployed field methods, considered the periods spent on various onboard ships offshore by the principal researcher in 2017, 2018, and 2020, before and during the early stages of the COVID-19 pandemic. This limited access limited the geographical boundaries of the study to offshore and port locations within Ghana and the Ivory Coast. In addition, vessels’ security operations were observed to understand how shipboard management ensured the safety and security of crew and cargo at various waypoints, offshore locations, and during port calls (seen in Fig. 2) regardless of the security level at any point in time.

The second and third phases of the study occurred mainly within February and May 2021 while ensuring that COVID-19 protocols had been adhered to. Thus, the deployed surveys were intended to elicit responses given by participants administered through interviews or questionnaires. Due to the ongoing health crises, all oral interviews and questionnaires were administered through various online portals, such as direct email listings. They were also shared with respondents on various social networks, including LinkedIn, Telegram, and Facebook. Respondents of both interviews and surveys were required to address why piracy and armed robbery are on the rise despite the COVID-19 pandemic. What measures are in place for tackling the problem? How would these measures promote maritime security post-COVID-19? Additional questions were addressed separately depending on whether the respondent formed part of the interviewed or survey group. These questions bothered their individual knowledge and experiences over the years for the former. The impact of maritime piracy in the subregion on economic cost will also be assessed while relying on secondary data gathered. The essence of this analysis is to consider the economic limitations of the growing maritime security concern projects on the AfCFTA implementation.

#### Target population and sample size

The target population selected in this study was considered based on three fundamental reasoning principles: familiarity, knowledge, and exposure to maritime security concerns. The peculiar nature of the study meant that the seafarer population was at the centre of the study. Additionally, the target population also considered other parameters, such as the operational domain, e.g., for offshore installations, such as mobile offshore drilling units (MODUs) and floating production storage, oil tanker carriers and offloading (FPSOs) units, vessels restricted to manoeuvrability near coasts, such as fishing vessels engaged in fishing or offshore exploratory vessels, and shore facilities with varying sets of critical marine personnel other than seafarer groups. This includes offshore operation managers, surveyors, remote operating vehicles (ROV) technicians, and project engineers.

Again, due to accessibility concerns, the target population sampled was narrowed to encompass personnel living or working within Africa’s maritime corridors. It, therefore, encompassed personnel on vessels’ researchers had corresponding voluntary investigators easily assigned at various stages of the study. Ghana remained the central focus of the field study in the GoG region due to its proximity and accessibility to researchers; hence, a select few maritime experts from key statutory maritime institutions were surveyed to help develop an understanding of trends. In addition, the ship crew and shipside marine professionals sampled were gauged over their experiences and concerns on the investigated issue. The sample size is therefore detailed in Table 1 below.

**Table 1:** Study sample chosen with respect to population target. *Source*: Field data

Targeted Population	Sample size	Actual population respondents
Ship Crew	80	60
Shipside Marine Professionals*	20	10
Statutory maritime institutions	5	2
Total	105	72

*Shipside Marine Professionals refers to marine professionals whose duties and work obligations place them temporarily onboard marine vessels and are not necessarily part of the ship. Examples include marine surveyors, superintendent of ship cargo, subsea engineers, ROV pilots, Client representatives, Offshore Construction Manager (OCM), and so forth.

The population sample chosen is shown in Table 1. The sample size comprised 100 respondents divided into three categories.

#### Sampling technique and ethical consideration

Purposive and quota sampling are used within the target population based on selected elements and time limitations placed on the research data gathering process. However, all respondents were informed of the intended use of the data provided. Therefore, the responses expressed could only be under the terms of unanimity. These sampling techniques of choice and terms of unanimity were essential to the urgency and intent of the study to help address the growing concern of maritime insecurity before, during, and after the COVID-19 pandemic.

### Data gathering

The data gathering process considered the three-phase design of the study in gathering the desired data for analysis over specific sets of time frames. The survey of seafarers, which marked the final stages of the study’s data gathering process, was carried out over the last three months in the first quarter of 2021. However, the field observations preceded the seafarer survey phase, thus commencing in 2017 and concluding in 2020. The first leg of the field observation focused on Ghana’s coast and offshore locations, while the second leg was within the port of Abidjan in March 2021. However, activities under the field observations were restricted to compliance with various COVID-19 protocols at the time. The interviews section crowned the data gathering process intermittently within three months concurrent with the online survey.

The instruments chosen for this research were questionnaires, field observations, and interviews for primary data gathering. The study also reviewed secondary data on the subject matter, including online news articles, electronic magazines, books, web articles, and journals. The instruments chosen and deployed for the primary data were various technology devices and platforms. Mobile phone calls and text platforms such as WhatsApp, Google Form, LinkedIn, and Facebook Messenger were utilised. Relying on these platforms was imperative in the era of the COVID-19 health crises, safety protocols, and travel restrictions witnessed in the first, second and third waves of the COVID-19 pandemic variant across the world.

The researchers could not travel to meet every seafarer worldwide, so the abovementioned tools were deemed appropriate to sample the selected population.

### Data analysis

**Fig. 3:**
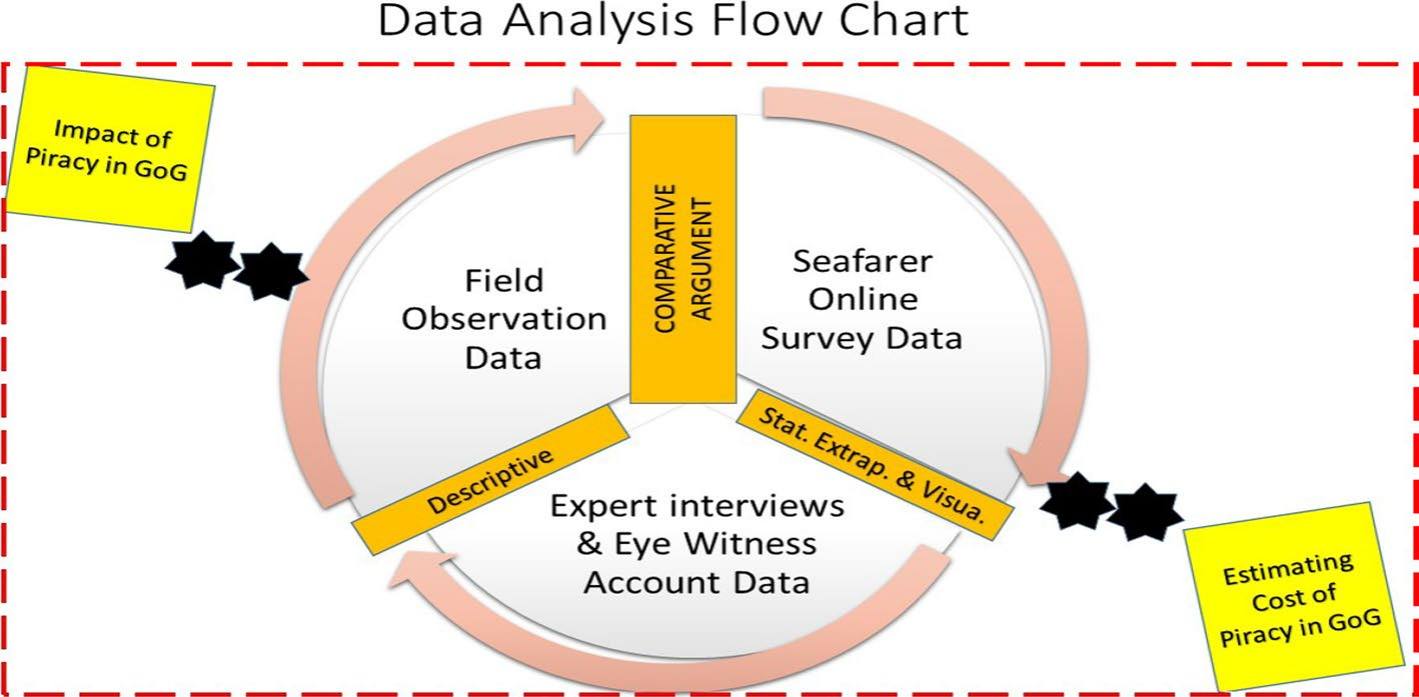
Analysis flow chart of the study. *Source*: Authors

Figure 3 shows the data analysis flow, divided into two criteria discussed in the subsections below.

#### The cooccurrence of maritime security concerns to maritime professionals and GoG nations

The data analysis in this study accounted for both quantitative and qualitative data generated under the three-phase design of the study via the three unique sets of instruments deployed. This analysis was done alongside the need to estimate piracy’s cost in the subregion. As shown in Fig. 3, the first stage of the three-phase design study, which generated data in line with direct field observations, was analysed descriptively while making comparative arguments, inferences, and postulations. The second stage of the analysis relied heavily on quantitative data generated in the survey. Therefore, analysis at this study stage used graphical presentations and tabular extrapolations of figures and composed descriptive narratives for observations on quantitative data that highlighted the study’s objective—based on respondents’ perceptions borne out of experiences. Finally, the third stage of the analysis process seeks to make postulations and inferences from qualitative data obtained via interviews. It does so by making comparative arguments against survey and field observation outcomes.

#### Estimating the cost of piracy to gog nations in Africa

Estimating piracy’s cost is complex and challenging (Bowden et al. 2010; General Insurance Research Organising Committee, GIRO: Sanders et al. 2010). Nonetheless, the final stage of analysis in this paper attempts to evaluate the cost of maritime piracy in Africa based on Bowden et al.’s (2010) approach, which attempted to calculate the global costs of maritime piracy. The largest insurance premiums rates related to piracy (war risk and K&R) will be multiplied by 90 per cent of the total ship traffic volume transiting the high-risk region of the GoG (approximately 547,500 ships, given that 1500 ships ply the route daily) in a stated period.

Given that ship routes are not static and are relatively encircled across oceans globally, except for specific locations connected by unique canals for various ships, each vessel route choice varies depending on its voyage plan and special charterer requirements. Therefore, in estimating the cost, we consider the assumption that a proportion of marine traffic constituting 10 per cent of ships relatively trading down south of Africa over the period succeeded in rerouting around the Cape of Good Hope from the south—away from the piracy hotspot, the West African maritime corridor towards the Indian Ocean.

Again, we assume another set of ships similarly routing from the north did the same rerouting through the Suez Canal away from the GoG area. Therefore, a deduction of 10 per cent of marine traffic was considered valid, like those rerouting south along the Cape of Good Hope. Of the 80 per cent marine traffic left, we further assume set of 10 per cent ships by virtue of port or flag state location and trade route, were not liable for insurance premiums in the war risk region. Hence, a figure for the total amount payable on war risk insurance and K&R insurance is assumed to have been purchased by a higher bound estimate of 70 per cent for ships routed through the GoG region. But, again, on the assumption that not all ships may purchase war risk and K&R insurance, we chose a lower bound estimate of 10 per cent—constituting all such ships uninsured with war risk insurance and K&R.

The analysis is further developed with supportive comparative arguments based on qualitative results from expert opinions, a technique relied on by Ellinor (2013), Lamptey and Sackey (2017), Sackey and Lamptey (2019), and the literature on ongoing AfCFTA implementation amid the COVID-19 crisis.

## Results

The results presented in subsections in this study are constituted by analysis and discussions based on outcomes from research interviews, field observations, survey interpretation, and estimation of the cost of piracy and implications for economic advancement in Africa.

### Reported outcomes from the respondents’ interviews

A total of five individual experts from the various statutory institutions with relevant maritime knowledge and background were the selection target for the interviews; however, two of the selected experts were from two institutions, namely, the Ghana Navy (GN) and the Ghana Maritime Authority (GMA), which were available for interviews in this study. The first, who is currently an active commander within the eastern naval command front of the Ghana Navy, has over 13 years of maritime law enforcement experience. The latter, who serves with the GMA as an active port state control (PSC) inspector in the regulatory capacity, also wielded excellent experience in port and coastal state requirements for ships in the GoG region. Together, their experiences and knowledge espoused should shed more light on the concerns in the region, as shared in the paragraphs below.

#### What role do regulatory authorities and shipowners play in situations of attacks on crew

According to resource persons, governmental efforts towards aiding ship crew in piracy confrontations ensure the safe release of ship crew from their captors. These efforts mostly hinge on successful military operations under articles 102, 103, 104, 105, 106, 107, and 111 of the United Nations Convention of the Law of the Seas, UNCLOS 1982. Likewise, West African nations’ governments do not negotiate with criminal syndicates whose goal is to continue perpetrating crimes to the detriment of their societies. Unfortunately, there are no guarantees in this single approach during military operations. Therefore, ship owners and insurers continue to assist diplomatically in paying ransoms in most situations where a successful release of the kidnapped crew occurred. Despite this drawback, military efforts are growing with the various collaborations implemented. The Nigerian Maritime Administration Support Agency (NIMASA) established the Deep Blue Project (DBP) for maritime security to combat piracy problems in the GoG region. Its operation focuses on the Nigerian anchorages forming part of the DBP jurisdiction. The setup also coordinates interagency information sharing between GoG nations regarding maritime security threats and concerns.

Again, focusing on recorded pirate cases in January, February, April, May, June, July, August, September, and November 2020 (also seen in Fig. 8 on page 15) and between January and March 2021, where 15 crew members were kidnapped, one recorded incident of crew death each while discussing with expert respondents. In addition, experts highlighted intensified naval patrols, growing regional collaborations, and international support to buttress the ongoing efforts. Thus, according to them, relying on Yaoundé Architecture for Maritime Safety and Security (YAMSS) resources, newer acquisitions of naval gadgets and surveillance assets across the various naval institutions in Africa have become possible within a short time. Unfortunately, the military operations in the region have suffered several fatalities (especially of the Nigerian navy) during hot pursuits with collateral damages, thus according to respondents. Therefore, several military exercises are taking place among regional naval institutions to boost naval capacity and capabilities. The latest to be conducted was in March 2021, hosted by the Ghana Navy, the ‘Obangame Express 2021’ (OE21), the largest maritime exercise ever conducted in Western Africa (GM Event 2021). Accordingly, government-led international efforts in the GoG area have seen the French and Italian navies make vessels available to support regional counterpiracy and maritime security efforts from early to mid-2020 (GM Event 2021). Another layer of interest in understanding the maritime security concerns examined is the eyewitness report detailed in subsequent paragraphs.

#### Interview of eyewitness respondents associated with piracy incidence in the GoG area

One respondent who unfortunately became a direct victim of a pirate attack near the coast of Equatorial Guinea in 2019 highlighted the concerns of the piracy menace among local seafarers of the region. The victim, a deck officer on board the vessel and his fellow crew, was the first to be attacked by pirates in the GoG. However, it is unclear if they raised the alarm when attacked. This situation notwithstanding, upon the hijack of their vessel, the pirates proceeded to use the hijacked vessel as a platform to aid in robbing other unsuspecting vessels plying the trade route of the Gulf area. With the Equatorial Guinea navy on high alert after receiving notification of pirate attacks near their coast over the period, it was in no time that the vessel was apprehended during the hot pursuit. All crew on board the hijacked vessel was subsequently taken into custody. The account suggested that the pirates on the hijacked vessel who boarded the unsuspecting vessel used the hijacked vessel and its crew as a decoy and were long gone before the navy’s arrival.

Subsequently, the owners of the hijacked vessel contacted the authorities of Equatorial Guinea with the necessary information and documentation, seeking the release of their crew and vessel. However, their request was denied. The diplomatic stalemate escalated, and soon the Nigerian government was involved. After several days into weeks in custody, the crew was finally released. The researchers’ focus on the aftermath of this single incident demonstrates the difficulties pirates’ victims encounter, indirectly due to the nature of the cross-border crime. Most respondents familiar with the incident that drew local attention among the West African seafaring community as an alumnus of the Regional Maritime University were sentimental in their responses and demanded proper surveillance and collaboration among stakeholders.

In the aftermath of the single incident referred to above involving the alumnus of the regional training institution, the researchers note that although the hijacked vessel used in committing the crime of piracy in the exemplary case here referred to was locally owned and operated by Nigerians through the nation’s cabotage laws. The circumstance does not negate any impending developments toward implementing cabotage laws being considered across the maritime industry in the region. Fundamentally, the regulation would ensure that regional and continental trading resulted in the overall growth of Africa’s economy. However, given that various data sources (including the ICCIBM reports) identify Nigeria as having the highest incidence counts in the region and being the home of the nationality most suspected pirates involved in hijacks and robbery in the region, it remains unclear to what extent these poorly regulated maritime corridors are exploiting the practice of cabotage in perpetrating maritime crimes.

### Outcomes from the online survey of seafarers

The reported results are presented in the section and categorised as seen below.

#### Demographic background of respondents

**Fig. 4:**
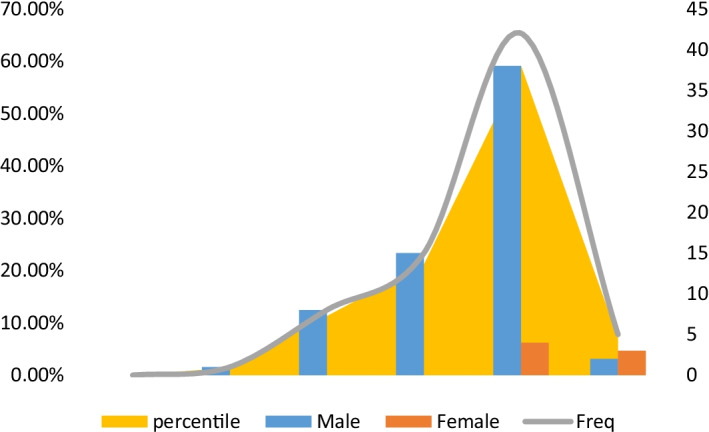
Age and gender of individuals sampled. *Source*: Authors

**Fig. 5:**
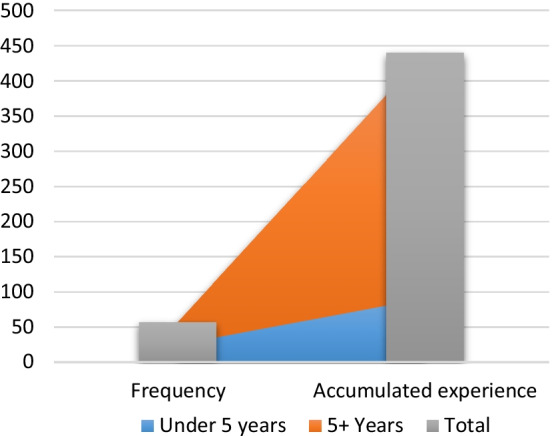
Professional years of experience. *Source*: Authors

A total of 105 respondents from the targeted maritime population in Table 1 were expected to be sampled; however, there were 72 actual respondents, of which 60 were of the ship crew. The background of the study examined respondents’ demographic data, considering the age ranges (seen in Fig. 4) and the level of professional experience (seen in Fig. 5). This was important to shedding light on their understanding and experiences of the incidents of maritime insecurities.

The ages of the female and male respondents are shown in Fig. 4. Of these, 59.15 per cent of respondents were within the 25 and 34 age range, and 21.15 per cent were within the age range of 35 and 44 years. No one was over 64 years old. A total of 7.04 per cent were also respondents aged between 18 and 24 years. The remaining 12.68% represented individuals belonging to the (45–54) and (55–64) year age groups.

These respondents were also rated for their experience level (seen Fig. 5). Those with under 5 years of experience amounted to 91 years. Those with 5( +)-years of experience in the maritime industry amounted to 349–440 years of experience. Most respondents indicated that they work in both onshore and offshore areas. Those whose experiences stretched to offshore areas engaged in the hydrocarbon industry on various field development projects across Africa. While most served onboard various heavy-lift and light construction vessels, MODUs, and FPSO units, others served onboard containers, roll-on roll-off (RORO) carriers, offshore support vessels (OSV), and platform supply vessels (PSV). These levels of experience in the view of researchers should strongly correlate with the views expressed by respondents who are empirical to the analysis.

#### Piracy and armed robbery experiences in the Gulf of Guinea

**Fig. 6:**
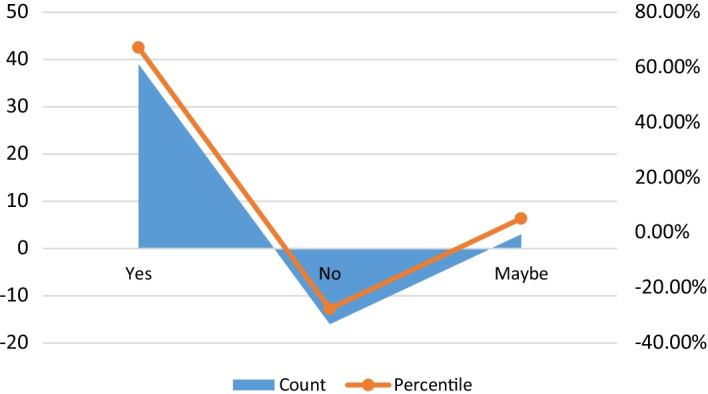
Respondent’s proximity to Piracy or high-sea armed robbery. *Source*: Authors

Researchers asked if the respondents or any close associates were aware of the piracy insecurity trends within the GoG and if they had any prior encounters with pirates or armed robbers in the area. The analysis of the responses given by respondents is shown in Fig. 6.

All respondents show an outstanding level of awareness. Figure 6 analysis reflects the responses of respondents for whom over 67.2 per cent indicated that they or their close associates had previous experience with pirates or armed robbery attacks within the GoG area. A total of 27.59 per cent indicated that they had no such experiences working within the GoG. However, 5.17 per cent were unsure if they had such encounters at any time at sea. Most suggested that their experiences were less than five years old, with most of their experiences within the last three years of the most recent decade. The outcomes seen here also reflect the rampant nature of attacks within the region and the levels of risk that seafarers are exposed to.

#### Challenges faced compared against the threat of piracy and armed robbery

**Fig. 7:**
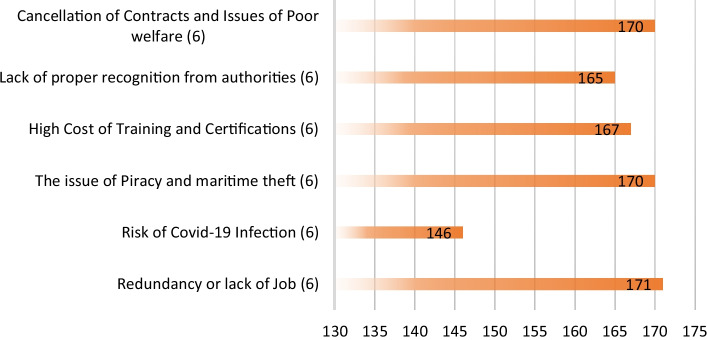
Respondents’ rating of the present challenges to the maritime industry of West Africa. *Source*: Field data

The seafaring community was then gauged for Respondents’ views on some significant issues they ranked higher as immediate concerns and why they felt so. For example, the health and travel restriction crises because of COVID-19 (Sackey et al. 2021a, b), the loss and lack of jobs, the lack of recognition, the high cost of training and certification, and the growing trend of piracy in the region were few identified as current problems faced by the seafaring and maritime community. The responses obtained are shown in Fig. 7.

The results from Fig. 7 show that the respondents ranked the issue of maritime piracy and armed robbery in the GoG second and on the same scale as the issues of contract cancellations that hit the industry, especially the upstream oil and gas sector, during the COVID-19 pandemic, resulting in the loss of jobs and an uncertain future. According to respondents, redundancy and lack of jobs amid the pandemic ranked higher because it created an environment of uncertainty for careers and families, as most had no other source of income. Respondents further indicated that none received welfare compensation from their employers upon being laid off. This was parallel with the losses most companies in the maritime sector were facing because of the cancellation of contracts fuelled by the travel restrictions implemented by nations within the region and worldwide. This claim is supported by the Public Interest and Accountability Committee (PIAC) report on Ghana as a case in point when examining the COVID-19 pandemic and associated shocks thus, according to Adom-Frimpong (2021). The 2020 PIAC report on Ghana’s upstream sector noted that signing new contracts, scheduling of projects earmarked for execution, and ongoing projects were mainly halted (Adom-Frimpong 2021).

**Fig. 8:**
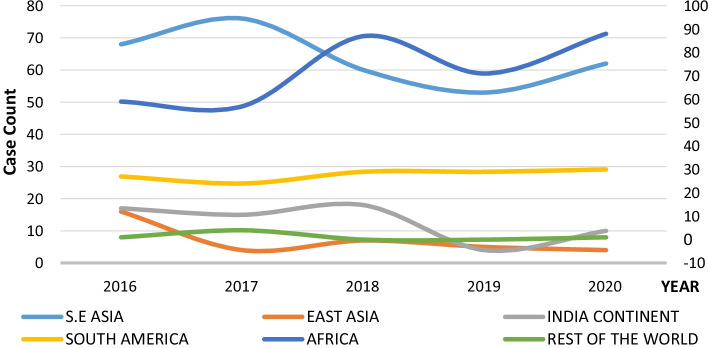
The Last Five Years’ Trend of Global Piracy and armed robbery based on ICC IBM data. *Source*: ICCIBM Report (2020)

However, piracy and armed robbery remained high during the period, as reported by the ICCIBM^2020^ report. Figure 8 shows the current piracy and armed robbery trends based on data from the IBM report between 2016 and 2020.

**Fig. 9:**
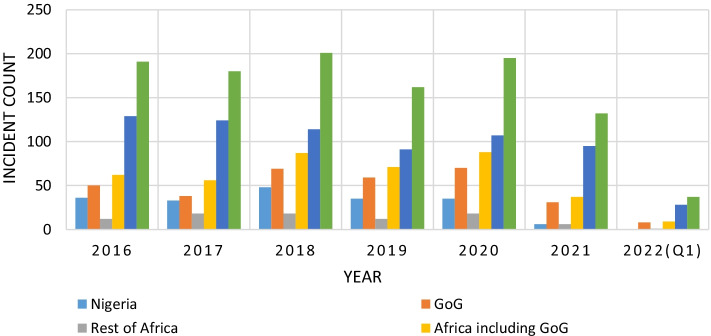
Regions of reported incidences. *Source*: ICC IBM Report (2020, 2021a, 2022)

Figures 8 and 9 show that the GoG area has witnessed a consistent rise in maritime security threats since 2016 and only dipped slightly in 2017 and 2019. Figure 9 also shows the subdivision of the data concerning incidences, helping identify the various hotspots.

Compared to incidences recorded in other areas across the world, this trend highlights the GoG area as a high-risk zone that ultimately affects the economic trading value of the region in terms of trade volumes and tariffs. From Fig. 8, the GoG area marked in orange can be classified as the hotspot. It is believed that piracy in the GoG is traceable back to the Niger Delta when attacks involved refined petroleum products carried in relatively small crafts, such as products and chemical tankers (United Nations Office on Drugs and Crime, UNODC, 2012).

Therefore, researchers discuss with expert respondents to understand the current problem and measures to curtail the situation. An expert respondent indicated that several measures by various navies are implemented across West Africa. Among the security measures identified as enforced are regular naval patrols. In addition, the joint naval patrols support these among West African nations. Under this scope of operations, the regional navies are occasionally joined by their international counterparts from Europe (e.g., France Navy), South America, and North America (US navy).

According to some respondents, expanding regional cooperation has also seen the Nigerian navy embark on a regional voyage to neighbouring West African nations, including Ghana and Gambia. There are also records of recent marine naval vessel acquisitions across the subregion. In addition, Ghana and Nigeria are restructuring their naval tactic even as they deliver newer assets such as speed boast. This and more continue to get implemented. This is detailed in subsequent sections.

#### The pre- and post-COVID-19 situation and its relation to insecurity status

**Fig. 10:**
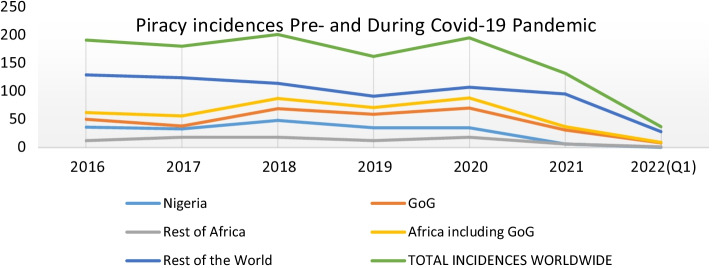
Regional Trends of reported incidences. *Source*: ICC IBM Report (2020, 2021a, 2022)

Concerning the pre, during and post-COVID-19 situational impact on maritime insecurity in the GoG region, the trends of the pre-pandemic situation are clearly depicted in Figs. 8 and 9 above (see also Fig. 10). Again, comparing the pre-pandemic and the pandemic (during phase) (i.e., between March 2020 until the date since an official end of the health crisis has not yet been declared despite having the pandemic under control relatively) eras, the pandemic projected a surge in piracy and armed robbery incident reporting. In addition, crimes in the GoG region, appear to have continued for some time into 2021 before dropping by the end of the 1st quarter of 2022. See Fig. 10 for further details.

According to ICCIBM (2021b), the first nine months of 2021 in the Gulf of Guinea region recorded 28 piracy and armed robbery incidents, compared to 46 in 2020. Crew kidnappings dropped to only one incident of kidnapped crew members during Q3 of 2021, compared to 31 crew members taken on five separate occasions in Q3 of 2020. Although the maritime insecurity trend amid the pandemic has not been consistent, they note that all the Q3 incidents in 2021 were against vessels at port anchorages. The average successful kidnapping location in Q3 2020 was approximately 100 NM from land (ICC IBM 2021b). While the slowing of reported incidences (see Fig. 10) has been widely acknowledged globally, the ICCIBM (2022) also suggests that this can be attributed to the efforts of the regional and international navies. According to them, collaborative work in the GoG region has reduced reported incidents from 16 in Q1 2021 to seven in 2022. Only a few months ago, the region became notoriously recognised as a hotspot. However, the IMB Piracy Reporting Centre urges continuous efforts to ensure piracy is permanently addressed within these highly risky waters (ICCIBM 2022).

### Field observation of ship crew experiences responding to piracy or armed robbery alerts

The results presented below capture the reporting of incidences and anticipated actions expected of crew members during distress alerts.

#### Reporting of incidence onboard

Respondents were asked about their experiences as ship crew and how they responded to piracy alerts broadcasted on the NAVTEX when they found themselves within the region of occurrence. They suggested that it is never a pleasant situation for anyone to experience piracy or its potential occurrence at any time. However, actions taken regardless of the distance between the broadcast location are consistent with the International Ship and Port Facility Security Code (ISPS Code) and designated security levels enforced upon the alert advice received under Safety of Life at Sea (SOLAS) 1974. According to deck officers of the Lewek Constellations and Seven Arctic, for which the lead researcher conducted the field studies, the ship captain, in collaboration with the ship security officer, had the mandate to raise the security level upon close examination of the facts under his discretion. This, however, depends on the unfolding of the situation.

Researchers observed that most ship owners and ship management companies encourage their operatives currently operating in the GoG region (thus designated as a piracy hot spot) to be on high alert regardless of the security levels implemented, whether offshore or port facility locations. Most occasionally, ISPS weathertight doors were kept closed on the instructions of the ship captain and ship security officer while creating a single entry and exit route for the onboard crew during transit from and to offshore facility locations and visiting crew when at port locations. The enhanced security situation naturally results in a restriction on mobility and operations.

However, the extreme vigilance per action of some ship captains, regardless of the security level implemented by the port facility locations in West African countries, is counterproductive, according to some port officials. For example, the attention of officials of Takoradi port was drawn to the fact that one of our platform-of-opportunity observer vessels berthed at the port Wharf had implemented an ISPS security level 2 designation while within the harbour. As a result, port officials called the captain on the radio, and it was demanded he resent the current security level to the lowest, for which he obliged.

#### Actions of crew counteracting piracy alerts

Respondents further indicated that whether a piracy threat is deemed real or not, the psychological impact from anxiety, uncertainty, constant anticipation, and fear of losing one’s life is a trauma that sits with the crew as they fight to implement security measures in anticipation. They suggested the constant practice drill of the ISPS Code’s security awareness training with designated duties for each crew member. According to them, under safety and security, emergency drills are a source of comfort in a difficult real-life situation for which outcomes are unpredictable.

Posing a vigilant lookout for any suspicious activities within the work vicinity within the GoG by strange vessels is a consented effort for the crew onboard the vessel, according to respondents, and is not limited to only the Officer of the Watch (OOW) or members of the marine deck crew. Immediately, any such observations should be reported to the ship’s bridge, possibly via radio. All these are measures recommended by the ICC IBM, as they warned ships operating in the GoG region of eminent attacks (ICCIBM 2021c).

It was incumbent on the crew to ensure the awareness of the various security protocols as per the ISPS code and ship security plans and means of raising the alarm or reporting such incidents no matter their role. This code includes familiarising oneself with the designated haven during ISPS emergencies. Such an occasion also demands crew lookout for one another, ensuring that less experienced guided individuals are safe. Respondents reiterated that it is not the vessel crew’s role to act as heroes during such attacks and therefore ensure they control their emotions, potentially resulting in a fatal adverse reaction. Therefore, a mental state of calm must always be from all crew members.

### Estimated cost of the piracy threat against the AfCFTA stimulating Africa’s maritime growth

According to ICCIBM (2021c), the first quarter of 2021 counted 38 pirate attacks worldwide, which directly threatened the lives of 45 crew members. Of these data, the farthest distance covered by pirates was 212 nautical miles that occurred within the GoG area, risking the life of 15 crew, thus suggesting the extent of threat coverage from the coastline into the high seas the level of capabilities acquired by these pirates in the region. In addition, various institutions provide K&R coverage at varying premium rates. For example, Steamship Insurance Management Services Limited (SIMSL 2015) provides a K&R premium up to a limit of 10 million US dollars. Similarly, the Swedish P&I Club (n.d.) sets a limit of 30 million US dollars per incident or event. Therefore, estimates of the premium utilisation over the recorded K&R incidence period across the maritime industry are given in Table 2 based on ICCIBM incidence data (ICCIBM 2020,2021a).

**Table 2:** Estimated cost of reported ransoms per premium rate to piracy event. *Source*: Authors

	2016	2017	2018	2019	2020	2021	
						Q1	Q2
Incidental Cost of Event per Premium	62	75	83	134	135	40	50
						90	
Max 10million USD	620,000,000.00	750,000,000.00	830,000,000.00	1,340,000,000.00	1,350,000,000.00	900,000,000.00	
Max 30million USD	1,860,000,000	2,550,000,000.00	2,490,000,000.00	4,020,000,000.00	4,050,000,000.00	2,700,000,000.00	

For the general cost of piracy in the GoG region, the data obtained and evaluated are presented in Table 3 based on the assumptions considered under the data analysis.

**Table 3:** Projected estimated cost of piracy in terms of insurance acquisition over the given period of study. *Source*: Based on ICC IBM data and premium of the Swedish club

	2018	2019	2020	2021 (Q1 &2)
Average vessel utilisation of the GoG area	547,500	547,500	547,500	279,000
Max K&R premium considered (USD) per Swedish P&I club	30,000,000	30,000,000	30,000,000	30,000,000
Kidnap & ransom surcharge	1.47825 × 10^13^	1.47825 × 10^13^	1.47825 × 10^13^	7.533 × 10^12^
Total insurance costs (if all ships purchased)	1.6425 × 10^13^	1.6425 × 10^13^	1.6425 × 10^13^	8.37 × 10^12^
Lower bound estimate (10%)	1.314 × 10^13^	1.314 × 10^13^	1.314 × 10^13^	6.696 × 10^12^
Upper bound estimate (70%)	1.14975 × 10^13^	1.14975 × 10^13^	1.14975 × 10^13^	5.859 × 10^12^

It is imperative to note that today’s maritime insecurities are inevitable, given that rerouting, delaying a voyage, or acquiring additional security measures in addition to insurance cover presents an additional cost to shipping operations in the region and worldwide. As AfCFTA implementation continues throughout the continents, there is great expectation for industrialising Africa. The industrialisation drive is to grow the trade volumes. These efforts ultimately will rely on local and regional interest in areas of maritime transport network infrastructure and the business of vessel services. Therefore, liner and tramp vessels are crucial to enhancing connectivity since they can deal with transporting large volumes of goods compared to trucks and road networks. These road networks are complemented by the rapid expansion of railway infrastructure connecting landlocked countries.

Maritime cabotage laws as implemented in Nigeria can therefore shape the future of Africa if adequately implemented across the continent. In contrast, efforts to eliminate pirate attacks in the region expanded catered to a possible shift, thus widening the GoG hotspot locations (ICCIBM 2021c) into lesser patrol areas along the African coast post-COVID-19. According to respondents, every effort implemented currently in the GoG area should consider a segmented sweep patrol field design collaboratively from every coastal nation to the coastline of Africa. Using the 6,000 km coastline of the GoG nations’ battle with the rising incidence of attacks as a learning curve, the situation should help develop a long-term maritime security administration to ensure Africa’s continuous sustainable trade and economic growth.

The designation of the GoG as a maritime security hot spot (ICCIBM 2021d) in the most recent time has an implied cost for shipping that directly reflects today’s rising inflations among West African nations as the global economic recovery continues. This excess cost tends to stifle all socioeconomic gains while leading to the collapse of small-scale businesses.

#### The practice of cabotage and local content laws and concerns for maritime security

For developing countries and regions with low economic capital and political power to a growing population, infrastructural deficits, high levels of illiteracy and unemployment, legislating for wealth appears to be the only viable solution to a highly competitive global market dominated by developed countries in addition to regular humanitarian aid.

The laws on local content in Africa today deal with concerns about getting local investors to favourably participate in the extractive sectors of natural resources in Africa while encouraging joint venture (JV) opportunities with potential foreign partners. In Ghana, the requirements seek to promote value addition and job creation, capacity building, and high-level acquisition of capital assets by indigenous operatives in the petroleum sector (Petroleum Commission Ghana, PC 2019). Such an effort is in line with AfCFTA’s goals for the continent. The lead researcher benefited from the occasion to observe the implementation of the laws in the oil and gas engineering service sectors from 2017 through 2020 on the OCTP (Offshore Cape Three Point) Sankofa Gye Nyame field development and the Jubilee Turret remediation projects. Deliberate efforts ensured that local Ghanaian crew members with the prerequisite seafaring documentation were absorbed and further trained into various roles onboard MODUs, Offshore Construction vessels, OCVs and FPSO units. The appointments encompassed low- and middle-level vessel management and operational responsibilities.

Although the larger chunked were evidently in low-level roles (such as rigging technician, able-bodied seaman, welders, motorman and occasionally cadets), the consistent efforts ensured the transfer of skills and exposure to real work scenarios. Evident this assertion, the researcher also observed a progressive manpower development onboard marine vessel *Polar Onyx* subsequently chartered by subsea construction company Deep Ocean Ghana. Thus, through consistent development of some Ghanaian crew while on the job training from 2018 to 2020, some of these individuals were promoted to mid-level management based on performance assessment. Others took on the operational roles of deck foreman and shift supervisor. It is essential to note here that most of the young seafarers in the African Seafaring community have on average high school and above the level of education and therefore are the industry’s future if adequate policies implemented are to capitalise on the development (Sackey et al. 2021a). These efforts highlight the economic benefit of the implemented LOC (local content) regulations. Cabotage laws are seen as a step further in this agenda.

The call to implement maritime cabotage laws continues to grow in Africa, although little has been done to ensure it becomes law across most African countries. For example, Nigeria successfully implemented the cabotage law in recent years and saw a rise of indigenes manning vessels operating in their territorial waters. One such vessel observed in a 2020-year operation was the anchor handling tow support vessel (AHTS) Seven Adaba of NigerStar 7, which assisted with barge towing between the port and offshore locations within Ghana. The successful operational model emulated can be under cabotage practice across Africa. Thus, they should be properly aligned with Economic community of West African states, ECOWAS and the AU trade protocols. The growing interest among countries suggests the need for the AfCFTA on maritime trade practices to incorporate a measured scheme of the cabotage principle under its framework for trade operations to ensure growth.

While these calls are noble in enhancing economic empowerment to locals in the region, the concerns of maritime insecurity have become apparent in relative terms, with the GoG region currently marked as a piracy hotspot, raising concerns of capacity security and insurance, financial, regulatory, and legal institutions in dealing with the challenge. Questions arise as to the capacity of local insurance companies to handle K&R claims. While Nigeria’s cabotage law remains a typical example of cabotage implementations, the high rate of pirate and armed attacks recorded as originating from Nigerian waters and high seas leaves much to be desired. The situation beckons with questions rather than answers—suggesting that any effort at implementing a cabotage law must first be grounded in an adequately regulated maritime space. However, researchers observe a rise in tramp shipping within Nigeria—constituted by tugboat towing barges stacked with container loads. They assert that as a result of the implementation of cabotage, this have become a common phenomenon across the waterways of Lagos and its surrounding while easing traffic flow on the various land road networks. However, the study observes some concerns related to safety in operation due to poor standards of some practitioners.

#### Potential role of fishing communities and artisanal fisherfolks against maritime insecurity

Two approaches have been identified in response to Africa’s age-old maritime piracy problem. First, the fight against insecurities in Africa today focuses on the reactionary approaches, thus concerning crew training in preparedness for potential security emergencies and national response through naval war assets, highlighted in the previous sections. Second, some experts have called on the need to ensure economic prosperity in fishing communities to curtail the vulnerability of individual artisanal fisher folk’s exposure to groups of organised crime syndicates. Such an approach is a proactive, indirect cause and effect measure, although it cannot easily be measured for successes or failures.

**Fig. 11:**
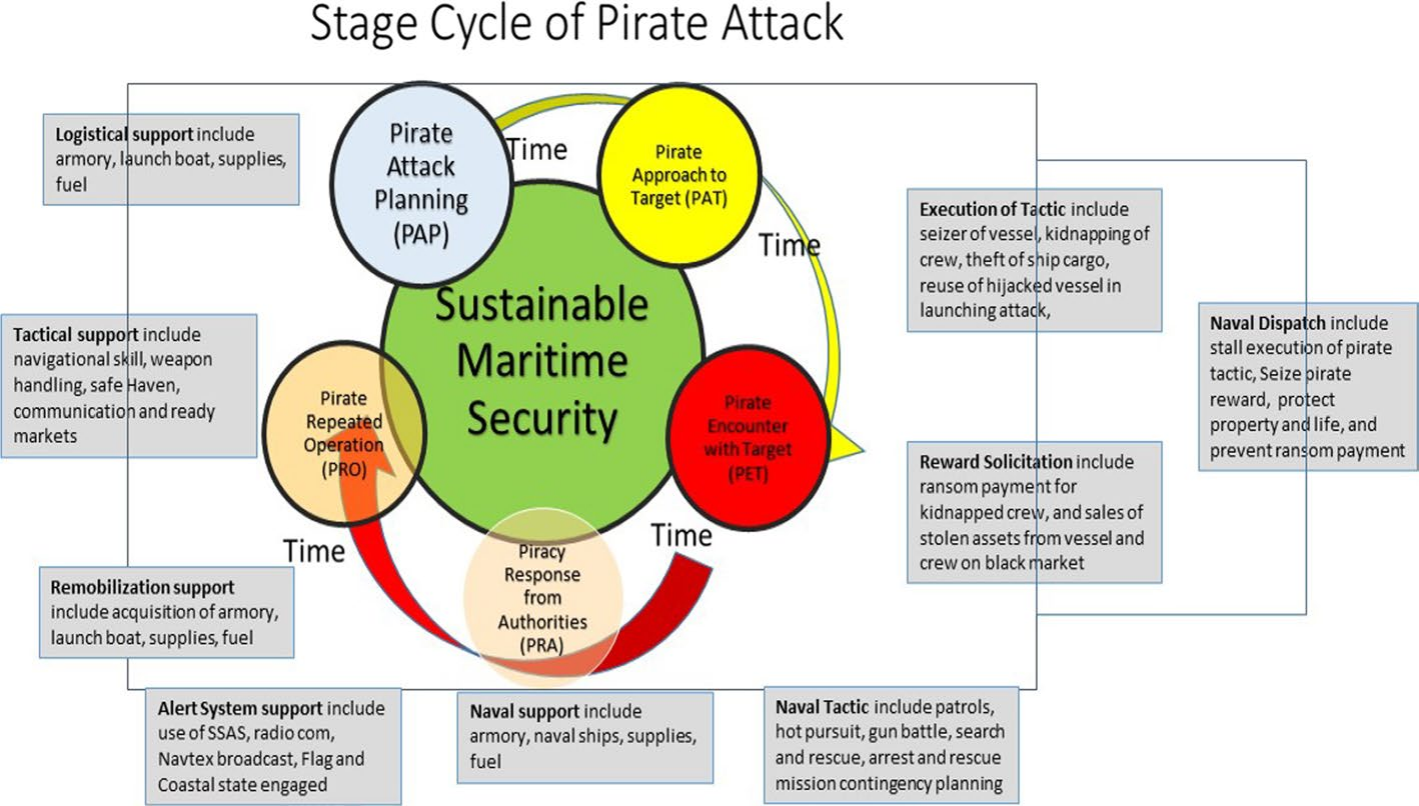
Cyclical growth stages of piracy in the Gulf of Guinea region. *Source*: Concept-based on field data analysis

During field observations of the various actors engaged in operations in the GoG area, the researchers observed four stages involved in a maritime *piracy (or armed robbery) cycle.*^2^ Thus, they all have various timeline lines, actors, defined requirements, and actions. This framework of the pirate operational stages depicted is shown in Fig. 11.

Figure 9 illustrates the flow of pirates’ conception and planning of an attack from the pirate attack planning (PAP) stage and proceeds to execute their operation by approaching the unsuspecting target vessel. At the pirate-approaching target (PA) stage, the situation becomes visibly apparent to the targeted vessel operators, who may raise the alarm through the Ship Security Alarm System (SSAS). The alert is subsequently broadcasted via NAVTEX to all vessels within the vicinity, flag state and the coastal states nearby, exercising the right to hot pursuit for the pirates of the pirate encounter stage (PET). Responses, in this case, can be of two outcomes, either successful or failure. The latter is usually the case. Hence, the pirate operations proceed to the next and final stage, called the pirate repeated operations (PRO) mode level. This period involves regrouping and strategizing—where needed—the acquisition of operational assets from proceeds. The final stage informs their next planned attack and the needed preparations.

Hence, there are five visible intercepts (see Fig. 11) by which these pirate attacks can be foiled along the growth cycle in the GoG region, thus, proactively within the given framework of time if all the needed resources are available. However, the first resource required to be proactive is an eye-on-sight approach that can rarely be achieved; given the wide range of the coastline, the few naval boats must be covered in regular surveillance. Therefore, researchers believe that the role of the fishermen and the fishing communities should be defined appropriately in this fight, thus allowing for the proper allocation of resources to enhance effective collaboration.

This model should follow a modified version of community policing but on the high seas. Thus, all fishing communities should be engaged in various forms of dialogues, education, and training. A successful outcome should result in forming watchdog groups that consist of naturally experienced fishers and slightly educated members who can assist in communicating or operating radio alert beacons. All local and international collaborations will yield poor results if the side-line of these local artisanal fishers continues. Thus, they are seen as less of the foremost stakeholders in providing a solution to reducing or eliminating the menace. Again, some amount from international financial aid received towards fighting piracy can be used in securing mobile technological alerts and communication systems that the local leaders can distribute to the operatives while at community landing beaches before any fishing expedition at sea.

Proper motivations such as financial or material incentives should accompany the proposal for any successfully reported alert that leads to a successful operation. In addition, there should be an open communication channel between the security apparatus and the operatives of the local fishing boats (LFBs) to ensure transparency and trust.

## Conclusions

The two-part study concludes by encouraging the need to implement novel but human-centred community-based approaches to improve the current state of maritime insecurity. This intervention is against the backdrop of data from the first quarter of 2021, indicating that the GoG area accounted for 43 per cent of all cases, including gunfire and hijackings. Moreover, hijacked fishing vessels primarily serve as mother vessels for further pirate operations (ICCIBM 2021c).

On the concerns of AfCFTA considerations and the need to boost economic recovery post-COVID-19 pandemic, successful implementation is expected to significantly increase the number of consumer goods and services exchanged across the length and breadth of Africa based on the various modes of transport currently available. This includes a rise in the shipment of goods across Africa along the seafront compared to truck shipment, highlighting the safety and security concerns of Africa’s major sea trading routes that need to be fortified against armed robbery and piracy (GM Event 2021). Furthermore, other economic advancement policies, such as the implementation of cabotage laws in the region, also stand to fail where piracy concerns are not addressed holistically. Moreover, the insecurity situation ordinarily continues to add to the shipping cost in terms of insurance premiums such as K&R coverage. Ironically, this makes shipment to the region more expensive than other areas in the world despite the low-income status of consumers. Therefore, every and any effort at averting the current trend is welcomed on the back of applicable regulation and security policy implementation within the region.

Today, these efforts leading to enforcement hinge on successful military operations under articles 102, 103, 104, 105, 106, 107, and 111 of the UNCLOS 1982, alongside the various MOUs established under regional protocols. While efforts at detriment and prevention through regular naval patrols have failed to stop perpetrators within the societies due to the share size of surveillance coverage required in the region being inadequate, the increasing support received from international partners appears to be making headway. Unfortunately, there are no guarantees to this singular military operations approach without the required intelligence of intended crimes. It is essential to reiterate here once more that local fishermen from these coastal communities are a critical stakeholder group that cannot be neglected in the fight against maritime insecurity. Therefore, devising a means to engage them remains crucial.

Herewith, the community police model is identified as one such approach. This model to be implemented by the national navies in the region could mimic the community policing model implemented by the Ghana government through police service. The unit should serve as ‘eye-on-sight’ surveillance for authorities of any suspected events at sea. The approach has the potential for regional implementation with the support of regional blocks. For the model to be effective, the select lead of the artisanal fisher folks requires training in addition to the general education of all their members in the individual fishing communities. They will require portable remote alert warning systems that are easily triggered. There should also be a reward system that motivates these fisher folks while providing the fisher folks feedback on every alerted hot pursuit. However, further studies are required to streamline its implementation approach on a pilot basis.

Given that the economically estimated piracy cost in the GoG region currently remains scant, as insufficient data exist, which is essential in evaluating the economic impact on the subregion and impact worldwide, a further study in this regard has been proposed based on the mathematical models by Besley et al. (2012) while considering all the factors and datasets relevant to establishing costing analysis.

These assessments of maritime insecurities are imperative because the study views maritime insecurity as inimical to the potential progress expected with implementing the AfCFTA, thus ensuring the transition from a consumer-led economy to manufacturing. The maritime cabotage law continues to gain steam among neighbouring nations in West Africa. Nigeria’s case highlights these challenges if implemented in poorly regulated maritime waters. Therefore, where continent-wide implementation of cabotage is proposed under AfCFTA, such a measure should have a laid-out framework. Thus, operations are adequately regulated in maritime shipping corridors. Economically, it is unclear to what extent maritime insecurity impacts the region amidst COVID-19. The potential challenges will only be visible post-COVID-19 when the economy is out of recession. Therefore, ending the rising piracy incidence and armed robbery in the GoG as soon as practicable is non-negotiable for all stakeholders involved in the area.

## Data Availability

All datasets in this study pertaining to survey and interviews responses are available; however, due to the confidential nature of the materials (including WhatsApp communications) cannot be disclosed at this time except for the pooled score set from the survey in excel format.

## Abbreviations

ACET: African Center for Economic Transformation
AfCFTA: African Continental Free Trade Agreement
AMCHAM: American Chamber of Commerce-Ghana
AHTS: Anchor handling towing support vessel
AU: African Union
CPU: Community Policing Unit
CRIMSON: The critical maritime routes monitoring, support, and evaluation
DBP: Deep blue project
EC: European Commission
ECOWAS: Economic Community of West African States
FPSO: Floating production storage and offloading unit
GDP: Gross Domestic Product
GIRO: General Insurance Research Organising Committee
GM: Great Minds
GMA: Ghana Maritime Authority
GN: Ghana Navy
GoG: Gulf of Guinea
GPS: Ghana Police Service
ICC IBM: International Chamber of Commerce International Maritime Bureau
ISPS: International Ship and Port state security code
JV: Joint ventures
K & R: Kidnap and Ransom
LFB: Local fishing boats
LOC: Local content laws
MODUS: Mobile offshore drilling unit
MOU: Memorandum of Understanding
NIMASA: Nigeria Maritime Administration support Agency
NM: Nautical Miles
OCM: Offshore construction manager
OCTP: Offshore Cape Three Point
OCV: Offshore construction vessel
OE21: Obangames Express 2021
OOW: (Navigating or Engineering) Officer Of the Watch
OSV: Offshore support marine vessel
PA: Pirate approaching target
PAP: Pirate attack planning
PC: Petroleum commission (of Ghana)
PET: Pirate encountered target
PIAC: Public Interest and Accountability Committee
PRO: Pirate repeated operation
PSV: Platform support marine vessel
RORO: Roll-on roll-off marine vessel
ROV: Remotely operated vehicle
SOLAS: Safety of life at sea convention
SSAS: Ship security alarm system
UNCLOS: United Nations Convention on Law of the seas
UNODC: United Nations Office on Drugs and Crime
YAMSS: Yaoundé Architecture for Maritime Safety and Security

## Footnotes

1 This paper, is Part Two of a set of two papers on maritime security. The first paper (Part One) is titled, “*Tackling Impact of Maritime Insecurity within the Gulf of Guinea Along With Promising Expansion Of Trade Coastal West Africa”.*

2 Piracy (or armed robbery) cycle defined is as the functional process of successfully organised pirate attacks that ensures that continuous attacks are possible with improvement on the previous at the so choosing of the pirates. The cycle defines the growth stages of the operations up to the ultimate desired outcomes of the pirates.
